# Investigating the Potential Influence of *TAS2R16* Genetic Variants and Protein Levels on Multiple Sclerosis Development

**DOI:** 10.3390/jpm14040402

**Published:** 2024-04-10

**Authors:** Greta Gedvilaite, Enrika Pileckaite, Ignas Ramanauskas, Loresa Kriauciuniene, Renata Balnyte, Rasa Liutkeviciene

**Affiliations:** 1 Neuroscience Institute, Lithuanian University of Health Sciences, LT-50161 Kaunas, Lithuania; greta.gedvilaite@lsmu.lt (G.G.); loresa.kriauciuniene@lsmu.lt (L.K.); rasa.liutkeviciene@lsmu.lt (R.L.); 2 Medical Faculty, Lithuanian University of Health Sciences, LT-50161 Kaunas, Lithuania; ignas.ramanauskas@stud.lsmu.lt; 3 Department of Neurology, Medical Academy, Lithuanian University of Health Sciences, LT-50161 Kaunas, Lithuania; renata.balnyte@lsmu.lt

**Keywords:** multiple sclerosis, *TAS2R16*, rs860170, rs978739, rs1357949, polymorphisms, TAS2R16 serum levels

## Abstract

The study aimed to investigate the association between the *TAS2R16* gene (rs860170, rs978739, rs1357949), TAS2R16 serum levels, and multiple sclerosis (MS). A total of 265 healthy control subjects and 218 MS patients were included in the study. Single nucleotide polymorphisms (SNPs) were tested by real-time polymerase chain reaction (RT-PCR). The serum concentration of TAS2R16 was measured using the ELISA method. Analyses revealed that the *TAS2R16* rs860170 TT genotype was statistically significantly less frequent in the MS group than in the control group (*p* = 0.041), and the CC genotype was statistically significantly more frequent in the MS group than in the control group (*p* < 0.001). In the most robust (codominant) model, the CC genotype was found to increase the odds of MS by ~27-fold (*p* = 0.002), and each C allele increased the odds of MS by 1.8-fold (*p* < 0.001). Haplotype analysis of the rs860170, rs978739, and rs1357949 polymorphisms showed that the C-C-A haplotype was associated with a ~12-fold increased odds of MS occurrence (*p* = 0.02). Serum TAS2R16 levels were elevated in the MS group compared to control subjects (*p* = 0.014). Conclusions: The rs860170, rs978739, and rs1357949 polymorphisms demonstrated that the C-C-A haplotype and elevated *TAS2R16* serum levels can promote the development of MS. These preliminary findings underscore the importance of specific genetic variants, such as rs860170, rs978739, and rs1357949, in MS risk. Additionally, elevated TAS2R16 serum levels in MS patients suggest a potential role in MS pathogenesis. These findings provide insights into the genetic and molecular mechanisms underlying MS and pave the way for personalized diagnostic and therapeutic strategies. Integrating genetic and serum biomarker data in MS research offers promising avenues for improving clinical outcomes and advancing precision medicine approaches in the future.

## 1. Introduction

Multiple sclerosis (MS) is a chronic inflammatory disease affecting the central nervous system (CNS). It is manifested by the inflammation and degeneration of myelin sheath, axons and the accumulation of demyelinating plaques within the brain and spinal cord [1]. This disease afflicts individuals in the age range of 20 to 40 years, and women are 2–3 times more likely to suffer from this disease than men [2]. The total number of people with the disease is about 2.8 million worldwide, and the prevalence varies greatly depending on the geographic location. According to 2020 data, the prevalence in Europe was 142.81 per 100,000 population in the 35 European countries studied, while the prevalence in Africa was 8.76 per 100,000 population [3].

The exact etiopathogenesis of this disease remains unclear. Immune mechanisms and inflammation are essential factors in the pathogenesis of MS. However, it is still unclear whether inflammation is the first event in a cascade of pathophysiologic processes, a secondary response to an as-yet-unknown infectious agent, or primary CNS degeneration. It is a multifactorial disease, meaning genetic and environmental factors influence disease development [4]. Nevertheless, risk genes play a crucial role in MS. In particular, genes of class II hold significance as they encode molecules responsible for presenting antigens to CD4+ and CD8+ T lymphocytes. At MS, there is a notable association with the class II variant HLA-DRB1*15:01, with an odds ratio (OR) of approximately 3. Conversely, HLA-A*02, a class I variant, decreased the risk of MS, with an OR of around 0.6. When HLA-A*02 and DRB1*15:01 are considered together, the OR increases to approximately 5. The genetic and epidemiological fields have traditionally operated separately [5,6,7]. Also, there is a *TAS2R16* gene, located on chromosome 7 at position q31.32, that encodes a member of type 2 receptor family 16 (TAS2R16). This receptor is responsible for the perception of bitter taste [8]. One of the analyzed single nucleotide polymorphisms (SNPs) in ourstudy (rs860170) is a missense variant when the C allele changes into T [9]. Other SNPs are noncoding -rs978739 is an upstream variant, which encodes the T allele replacing C [10], and in rs1357949, the A allele replaces G [11]. These SNPs have been chosen due to the common genetic variability of *TAS2R16* [12]. Most human bitter taste receptors (TAS2Rs) have already been detected in the CNS, implying that they must play an important role in brain homeostasis and neuroinflammation [13,14].

The potential role of TAS2R in regulating ATP-binding cassette transporters (ABCs) and neuroprotective substances is still controversial. Nevertheless, TAS2R has been highlighted as a target for bitter therapeutics with neuroactive effects against various CNS disorders [15]. Although many TAS2R ligands, such as quinine and noscapine, are lipophilic and cross-biological membranes, ABC transporters also transport others. However, the question of whether they can also modulate the function of ABC transporters or exert other biological effects mediated by the activation of their receptors represents a new paradigm in brain research. Recent evidence suggests that extraoral activation of some TAS2Rs, such as TAS2R4, TAS2R16, and TAS2R38, prevent inflammatory responses [16,17]. Neurons in several regions of the rodent brain, including the hypothalamus, brainstem, and cortex, express not only TAS2R but also the intracellular taste signaling molecules α-gustducin, PLCβ2, and TRPM5 [18,19]. Although cerebral neurons responding to sweet and bitter tastes and astrocytes use similar signal transduction pathways, TAS2R recognition of pathogenic, toxicogenic, and microbial molecules in the brain is critical for preventing neuroinflammatory responses and for immune system functions under normal conditions [14,20,21,22].

Accordingly, impaired signaling or downregulation of key components of the taste signaling pathway has been associated with cellular damage due to increased oxidative stress and neuroinflammation [14,22]. These findings suggest a possible link between polymorphisms of this gene and MS. Therefore, this study aims to investigate the role of TAS2R16 serum levels and *TAS2R16* (rs860170, rs978739, rs1357949) gene polymorphisms in patients with MS in Lithuania.

## 2. Materials and Methods

The study received approval from the Kaunas Ethics Committee for Biomedical Research at the Lithuanian University of Health Sciences (No. BE-2-/102). The study was performed at the Laboratory of Ophthalmology, Neurosciences Institute, and Department of Neurology within the University Hospital of Lithuanian Health Sciences. The subjects who took part in the study provided written informed consent.

### 2.1. Study Subjects

Study subjects were divided into two distinct groups: patients diagnosed with MS (*n* = 218) and healthy subjects (*n* = 256). Patients with other systemic diseases (such as diabetes mellitus, oncologic diseases, systemic tissue disorders, chronic infectious diseases, autoimmune diseases, and conditions after organ or tissue transplantation), opacities of the optical system, or poor quality of fundus photography were excluded from this research. The diagnosis of MS was confirmed using the 2017 diagnostic criteria, which relied on clinical symptoms/relapses, Magnetic Resonance Imaging (MRI) findings of the brain and/or spinal cord with typical demyelinating lesions (according to MAGNIMS (Magnetic Resonance Imaging in MS) criteria), and positive oligoclonal bands (OCBs) in cerebrospinal fluid (CSF) [4,23]. The demographic factors of the patients in the study MS and the control group—age and gender—were evaluated in the study.

### 2.2. DNA Extraction and Genotyping

Blood samples were collected in K_2_EDTA tubes to extract DNA. Once all venous blood samples were collected, genomic DNA extraction was performed using the salting-out method with sodium chloride. The salting-out method is based on collecting cells using centrifugation, their suspension with a buffer solution, degradation of cell membranes with lysis reagents, deproteinization by proteinase K, nucleic acid separation with chloroform, and precipitation of DNA with 96% ethanol. Genotyping of *TAS2R16* (rs860170, rs978739, rs1357949) was conducted using the real-time polymerase chain reaction (RT-PCR) method. The genotyping was performed with the Step One Plus real-time PCR system (Applied Biosystems, Chicago, IL, USA) and TaqMan^®^ SNP genotyping assays (Thermo Scientific, Waltham, MA, USA), following the manufacturer’s protocol. The Allelic discrimination program was used during the RT-PCR. The program determined the individual genotypes of each SNP based on the fluorescence intensity of the different detectors (VIC and FAM). To ensure consistency, 5% of randomly selected samples were repeatedly genotyped for all three SNPs to confirm the same rate of genotypes from the first and repeated genotyping.

### 2.3. Sample Preparation for Serum Levels Measurement

For protein concentration measurement, blood from the participants was gathered in tubes without any added anticoagulant. After the blood collection, the tubes with blood were left at room temperature for 30 min to clot. Later on, the clot was eliminated by centrifuging at 1900× *g* for 10 min in a refrigerated centrifuge. After centrifugation, the supernatant—blood serum was kept in a freezer at −20 °C.

### 2.4. Enzyme Immunoassay

TAS2R16 serum levels were measured in duplicates in 20 control subjects and 20 patients with MS. This analysis was made out by ELISA method (enzyme-linked immunosorbent assay) using the Abbexa Human Taste Receptor Type 2 Member 16 (TAS2R16) ELISA kit (Abbexa LTD; Cambridge, UK). TAS2R16 serum levels standard curve sensibility range: 0.312–20 ng/mL, sensitivity < 0.1 ng/mL. The assay was conducted according to the manufacturer’s instructions, and the optical density was measured at 450 nm by the microplate reader (Multiskan FC Microplate Photometer, Thermo Scientific. Waltham, MA, USA). Each serum level of the TAS2R16 was calculated using the standard curve. 

### 2.5. Statistical Analysis

Statistical analysis was conducted using Statistical Package for the Social Sciences, version 29.0 for Windows (SPSS for Windows, Inc., Chicago, IL, USA). The frequencies of genotypes and alleles were expressed as percentages. To assess the normality of data distributions, such as age and gender, the Kolmogorov–Smirnov test was applied. It was determined that characteristics did not conform to normal distribution criteria. Therefore, the following descriptive statistical characteristics, including median and interquartile range (IQR), were used. The Chi-square (χ^2^) test was used to compare the polymorphism distribution of the *TAS2R16* (rs860170, rs978739, rs1357949). In binary logistic regression analysis, where analysis was adjusted by gender, ORs were estimated with a 95% confidence interval (CI) of MS considering inheritance patterns and combinations of genotypes. The optimal genetic model was selected based on the Akaike information criterion (AIC). Thus, the optimal genetic model was with the lowest AIC value. A statistically significant difference was found when the significance level < 0.05. *TAS2R16* haplotype association analysis was conducted separately for the patients with MS and the control groups. We used the online SNPStats website for haplotype analysis (https://www.snpstats.net/snpstats/ (accessed on 13 December 2022)). Associations between haplotypes and MS were calculated using logistic regression and presented as ORs and 95% CI. All haplotypes with less than 1% frequencies were grouped and labeled as “rare” haplotypes. A two-sided test with a value less than 0.05 was determined to be statistically significant. Finally, the Mann-Whitney U test was used to compare TAS2R16 serum concentrations across different groups.

## 3. Results

The study included 483 Lithuanian individuals in total. They were divided into two groups: a control group (*n* = 265) and an MS group (*n* = 218). For these groups, genotyping of SNPs *TAS2R16* rs860170, rs978739, and rs1357949 was performed. The control group consisted of 96 males (36.2%) and 169 females (63.8%), while the MS group consisted of 107 males (49.1%) and 111 females (50.9%), *p* = 0.004; thus, the subsequent analysis was adjusted by gender. The median age of the control group was 36 years. The median age of the group with MS was 38.4 years. The demographic data are shown in Table 1.

After analyzing the distribution of SNP genotypes and alleles, we found that the TT genotype of the *TAS2R16* rs860170 gene was statistically significantly less frequent in the MS group than in the control group (28.9% vs. 37.7%, *p* = 0.041). In comparison, the CC genotype and C allele were statistically significantly more frequent in the MS group than in the control group (7.8% vs. 0.4%, *p* < 0.001; 39.4% vs. 31.3%, *p* = 0.008, respectively) (Table 2).

Analysis of *TAS2R16* genes rs978739 and rs1357949 did not yield statistically significant results (Table 3).

Binary logistic regression analysis revealed that TT + CC genotypes compared with CT genotype is likely to be associated with 1.5-fold decreased odds of MS occurrence (OR = 1.491 (1.016–2.189); *p* = 0.041), while CC genotype, compared with CT + TT, is associated with 22.3-fold increased odds of MS occurrence under the recessive model (OR = 22.328 (2.947–169.181); *p* = 0.003). Under the codominant model–the CC genotype increases the odds of MS occurrence by 27 times (OR = 26.984 (3.504–207.790); *p* = 0.002). Also, it was found that each C allele is associated with 1.8-fold increased odds of MS occurrence (OR = 1.792 (1.267–2.533); *p* < 0.001) (Table 4). 

Analysis of *TAS2R16* genes rs978739 and rs1357949 did not yield statistically significant results (Table 5).

*TAS2R16* gene rs860170 CC genotype and C allele were more frequent in females with MS than in the control group females (9% vs. 0.6%, *p* < 0.001; 40.1% vs. 30.2%, *p* = 0.010, respectively) (Table 6).

Analysis of *TAS2R16* genes rs978739 and rs1357949 in the female group did not yield statistically significant results (Table 7).

Binary logistic regression analysis was performed in the female group. It revealed that rs860170 CC genotype is likely to be associated with 21-fold increased odds of MS occurrence in females under the codominant model (OR = 21.250 (2.607–173.207). *p* = 0.004). While under the recessive model, CC is associated with ~17-fold increased odds of MS occurrence in females (OR = 16.634 (2.098–131.870); *p* = 0.008). Also, each C allele increases the odds of MS occurrence by two times (OR = 1.995 (1.259–3.160); *p* = 0.003) (Table 8).

Binary logistic regression analysis of *TAS2R16* genes rs978739 and rs1357949 did not yield statistically significant results in females (Table 9).

We found statistically significant differences between MS and control group when analyzing *TAS2R16* gene rs860170 polymorphism genotypes (TT, CT, and CC) distribution in males (20%, 64.5%, and 6.5% vs. 33.3%, 66.7%, and 0%, *p* = 0.036) (Table 10).

Analysis of *TAS2R16* genes rs978739 and rs1357949 did not yield statistically significant results in males (Table 11).

Binary logistic regression analysis of *TAS2R16* gene rs860170, rs978739, and rs1357949 did not reveal statistically significant results in males, either (Table 12).

We performed a haplotype association analysis of *TAS2R16* rs860170, rs978739, and rs1357949 in patients with MS compared with a control group. Statistical analysis of MS and control group showed that individuals carrying haplotype C-C-A of SNPs rs860170, rs978739, and rs1357949 were associated with a 12.5-fold increased odds of MS occurrence (OR = 12.51 (1.59–104.12); *p* = 0.020) (Table 13). 

Haplotype analysis of MS and *TAS2R16* rs860170, rs978739, and rs1357949 showed no statistically significant results within groups by sex (Table 14 and Table 15).

**Figure 1:**
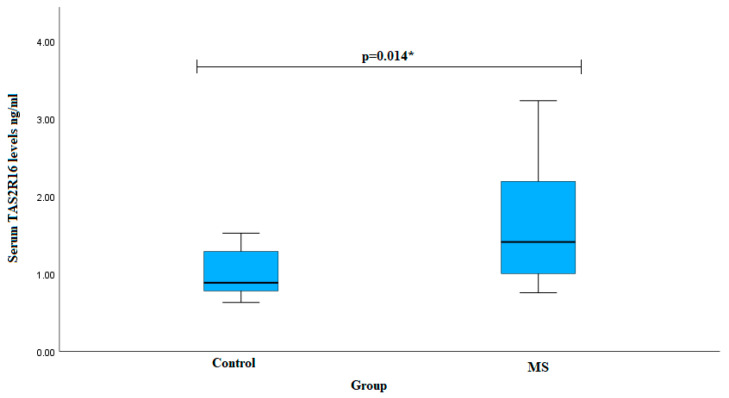
Serum TAS2R16 levels in MS and control groups. * The Mann-Whitney U test was used.

We assessed serum TAS2R16 levels in both patients with MS and subjects in the control group. The analysis revealed that the MS group exhibited higher TAS2R16 serum levels in comparison to control subjects (median (IQR): 1.412 (1.234) ng/mL vs. 0.886 (0.623) ng/mL, *p* = 0.014) (Figure 1).

## 4. Discussion

We studied the single nucleotide polymorphisms *TAS2R16* rs860170, rs978739, rs1357949, and TAS2R16 serum levels in MS patients and healthy individuals. Our main results revealed that *TAS2R16* rs860170 in the most robust (codominant) model, the CC genotype was found to increase the odds of MS by ~27-fold. In a sex-specific analysis of this polymorphism, the CC genotype increased the likelihood of MS in females as much as 21-fold in the most robust (codominant) model. Haplotype analysis of the rs860170, rs978739, and rs1357949 polymorphisms showed that the C-C-A haplotype was associated with a ~12-fold increase in the likelihood of disease occurrence.

In our research, for the first time, we analyzed rs860170, rs978739, rs1357949, and TAS2R16 serum levels in MS. However, there are no data in the scientific literature on the association between the *TAS2R16* rs860170, rs978739, and rs1357949 gene polymorphisms analyzed in this work and MS; there are studies analyzing the influence of these polymorphisms on other diseases, which development may be associated with inflammation/autoimmune diseases and other factors. For example, in 2017, Barontini and co-authors studied the associations of *TAS2R16* rs860170, rs978739, rs1357949, rs1525489, rs6466849, rs10268496 gene polymorphisms with colorectal cancer. Considering the importance of inflammatory responses in the development of colorectal cancer, scientists group investigated whether polymorphic variants of the gene *TAS2R16* could influence the risk of developing this neoplasia, suggesting that the role of *TAS2R16* may be related to the regulation of chronic inflammation. Although the polymorphisms studied in this work were not statistically significantly associated with colon cancer, the TT genotype of polymorphism rs1525489 was found to be associated with an increased risk of rectal cancer (OR = 1.62 (1.06–2.47); *p* = 0.007) [12]. In another study, Campa and co-authors 2012 examined the *TAS2R16* rs860170 and rs978739 gene polymorphisms and their associations with aging in humans. A statistically significant association was found between the rs978739 polymorphism genotype AA and longevity (*p* = 0.001). Also in this study, haplotype analysis of the *TAS2R16* polymorphisms rs1357949, rs6466849, rs860170 and rs978739 revealed that the haplotype (rs1357949-rs6466849-rs860170-rs978739: T_A_A_G) is associated with longevity (OR = 0.74 (0.56–0.98); *p* = 0.033). Given the importance of diet to longevity, genetic variation in taste receptors may directly affect aging by regulating food choices across the lifespan. One hypothesis suggests that polymorphisms in the *TAS2R16* gene may alter the perception of bitter taste, making people more likely to choose foods rich in salicin, a bitter compound with anti-inflammatory properties, thus improving quality of life [24].

Conversely, new evidence strongly suggests that taste genes have a significantly wider impact on human health. *TAS2R* family genes express membrane taste receptors in neuroendocrine cells of the digestive organs. These cells regulate various vital functions, including appetite, satiety, gastrointestinal epithelial cell proliferation, and many others important for longevity [24]. A similar study was conducted in 2019 by Malovini and co-authors. The *TAS2R16* rs978739 polymorphism was investigated, but this study found no statistically significant results between this polymorphism and longevity [25].

It has been identified that *TAS2R38* genetic variability haplotypes have a potential link to colorectal cancer (CRC) risk in two distinct Caucasian populations [26]. In contrast, SNPs within *TAS2R38* and *TAS1Rs* were found to be associated with an increased gastric cancer risk in the Korean population [27,28]. Scientists have examined the genetic variability of the *TAS2R16* gene, whose encoded receptor selectively connects with salicin, a natural anti-inflammatory substance very similar to aspirin [29,30]. Chronic inflammation is one of the strongest risk factors for colorectal cancer, mainly arising from diseases that trigger a continuous inflammatory response, such as ulcerative colitis and Crohn’s disease [31]. *TAS2R16* allelic variants and colorectal cancer risk, analysis for country of origin, authors found in the Lithuanian and the Spanish sub-populations a tendency for individuals with at least 1 C allele of the rs1525489 polymorphism to have an increased risk of developing colorectal cancer (*p* = 0.047 and *p* = 0.051, respectively). Nevertheless, after applying Bonferroni’s correction, the observed associations did not retain statistical significance. One plausible hypothesis to account for this phenomenon is that the identified SNP might interact with a lifestyle variable or a dietary practice, factors that were not included in our analysis. Alternatively, it is important to consider that the observed disparity could potentially be attributed to random statistical fluctuations. [12]. Schembre and their research team conducted a study investigating two *TAS2R16* polymorphic variants (rs846672 and rs846664) concerning the risk of developing colorectal adenoma. The rs846664 SNP displays monomorphism in Caucasians and, therefore, was not typed in this study, while rs846672 is in complete LD with rs860170, which was used in the present study (r2 = 1 in European HapMap Ceu). Upon conducting separate analyses for colon and rectal cancer, the researchers identified a correlation between the minor allele of rs1525489 and elevated susceptibility to rectal cancer specifically. This association was also shown to have a marginal/borderline statistical significance even after undergoing Bonferroni’s correction (*p* = 0.0071) [32]. 

As we mentioned earlier, there was no data in the scientific literature between the polymorphisms studied in this work and MS. Still, after analyzing the studies conducted by the researchers, the importance of the *TAS2R16* gene in the control of inflammatory responses and its role in the immune response was established. The association between the *TAS2R16* gene and MS may be attributed to the chronic inflammatory nature of the disease, where the patient’s immune system targets and damages the myelin sheath. Other inflammatory diseases, such as periodontitis, are characterized by inflammatory reactions resulting in lesions. This condition presents severe chronic inflammation leading to the destruction of tooth-bearing tissues and eventual tooth loss if left untreated. Human gingival fibroblasts, integral to the gingival connective tissue, play a role in perpetuating inflammation through the secretion of inflammatory cytokines in patients with periodontitis.

Studies revealed that gingival fibroblasts express 22 TAS2R subtypes, of which TAS2R16, TAS2R31, TAS2R38, TAS2R39, and TAS2R43 are the most abundantly expressed. To elucidate the function of these receptors in gingival fibroblasts, agonists that bind to the most highly expressed receptors were used. In this study, *TAS2R16* was most responsive to the agonist salicin and inhibited lipopolysaccharide (LPS)-induced release of proinflammatory cytokines (IL-6 and IL-8). Stimulation of TAS2R16 also reduces LPS-induced neutrophil recruitment, which is likely due to the reduced release of cytokines, particularly IL-8 [33]. Such a reduction in cytokine and neutrophil secretion could protect tissue damage and help control inflammation, and TAS2R family receptors could be used as a potential target for the treatment of MS. As mentioned earlier, infectious mononucleosis may play a role in the onset and development of MS [34,35]. It has also been observed that Epstein–Barr virus (EBV) may be one of the most important factors in the development of chronic periodontitis [36]. As both MS and chronic periodontitis involve acute inflammation and are associated with infectious mononucleosis, researchers explored potential connections between these conditions. In the study by Sheu et al., findings revealed that women with MS were 1.86 times more likely to develop chronic periodontitis (OR = 1.86, 95% CI: 1.39–2.48). However, no statistically significant associations were observed among men in relation to these diseases [37]. The association of the TAS2R16 receptor with the regulation of inflammatory responses in previous studies may indicate the importance of the TAS2R16 receptor in MS, especially since there is evidence that TAS2R16 receptors are also detected in the CNS [38,39]. This is because MS patients suffer from various symptoms, such as impaired motor functions, muscle weakness, and severe fatigue, which may make daily activities more difficult, which in turn may cause patients to have poorer oral hygiene and be more susceptible to periodontitis [40].

Also, it is essential to consider the possible implications of elevated TAS2R16 levels in the context of MS. The higher serum levels observed in our MS cohort could indicate a role for TAS2R16 in immune responses or inflammation, both of which are key elements in the pathogenesis of MS. Future research should aim to elucidate the precise mechanisms by which TAS2R16 may contribute to MS susceptibility or progression. 

The strength of the work: to our knowledge, *TAS2R16* gene (rs860170, rs978739, rs1357949) polymorphisms and TAS2R16 serum levels for the first time was evaluated in patients with MS.

Several limitations of the present study must be considered. The sample size for analysis of *TAS2R16* gene (rs860170, rs978739, rs1357949) polymorphisms and TAS2R16 serum concentration levels was rather limited and too small to reach the desired power setting. Unfortunately, our study does not incorporate clinical features such as EDSS scores, disease duration, MS phenotype, and other relevant clinical factors. Also, our current study lacks data on potential *TAS2R16* genetic variation in relation to lifestyle and dietary choices of MS patients. However, future studies will be conducted to explore these aspects in greater detail. Moreover, the study population consisted of individuals from a specific geographic region or ethnicity, which may limit the generalizability of our results to broader populations. Furthermore, it is important to note that this study is preliminary and requires confirmation in larger cohorts.

## 5. Conclusions

The rs860170, rs978739, and rs1357949 polymorphisms demonstrated that the C-C-A haplotype and elevated TAS2R16 serum levels can promote the development of MS. These preliminary findings underscore the importance of specific genetic variants, such as rs860170, rs978739, and rs1357949, in MS risk. Additionally, elevated TAS2R16 serum levels in MS patients suggest a potential role in MS pathogenesis. These findings provide insights into the genetic and molecular mechanisms underlying MS and pave the way for personalized diagnostic and therapeutic strategies. Integrating genetic and serum biomarker data in MS research offers promising avenues for improving clinical outcomes and advancing precision medicine approaches in the future.

## Figures and Tables

**Table 1:** Demographic indicators of the subjects.

Characteristic		Group		
		MS Group	Control Group	*p*-Value
Gender	Females, *n* (%)	111 (50.9)	169 (63.8)	0.004 *
	Males, *n* (%)	107 (49.1)	96 (36.2)	
Age median (IQR)		38 (15.25)	36 (27.5)	0.568 **

Abbreviations: *p*-value—significance level; * The Chi-square (χ^2^) test was used; ** The Mann-Whitney U test was used.

**Table 2:** Frequency of genotypes and alleles of the *TAS2R16* rs860170 polymorphism in patients with MS and control group subjects.

SNP	MS Group *n* (%) (*n* = 218)	Control Group *n* (%) (*n* = 265)	*p*-Value
Genotype			
TT	63 (28.9) ^1^	100 (37.7) ^1^	<0.001
CT	138 (63.3)	164 (61.9)	
CC	17 (7.8) ^2^	1 (0.4) ^2^	
Total	218 (100)	265 (100)	
Allele			
T	264 (60.6)	364 (68.7)	0.008
C	172 (39.4)	166 (31.3)	

^1^ *p* = 0.041 (TT vs. CT + CC); ^2^ *p* < 0.001 (CC vs. TT + CT). Abbreviations: SNP—single nucleotide polymorphisms; *p*-value—significance level; The Chi-square (χ^2^) test was used.

**Table 3:** Frequency of *TAS2R16* rs978739 and rs1357949 genotypes and alleles in control subjects and MS patients.

SNP	MS Group *n* (%) (*n* = 218)	Control Group *n* (%) (*n* = 265)	*p*-Value
*TAS2R16* rs978739			
Genotype			
TT	104 (47.7)	142 (53.6)	0.222
CT	99 (45.4)	100 (37.7)	
CC	15 (6.9)	23 (8.7)	
Total	218 (100)	265 (100)	
Allele			
T	307 (70.4)	384 (72.5)	0.484
C	129 (29.6)	146 (27.5)	
*TAS2R16* rs1357949			
Genotype			
AA	102 (46.8)	118 (44.5)	0.884
AG	94 (43.1)	119 (44.9)	
GG	22 (10.1)	28 (10.6)	
Total	218 (100)	265 (100)	
Allele			
A	298 (68.3)	355 (67.0)	0.651
G	138 (31.7)	175 (33.0)	

Abbreviations: SNP—single nucleotide polymorphisms; *p*-value—significance level; The Chi-square (χ^2^) test was used.

**Table 4:** Binary logistic regression analysis of *TAS2R16* rs860170.

Model	Genotype/Allele	OR (95% CI)	*p*-Value	AIC
Codominant	CT vs. TT CC vs. TT	1.336 (0.906–1.969) 26.984 (3.504–207.790)	0.144 0.002	645.637
Dominant	CC + CT vs. TT	1.491 (1.016–2.189)	0.041	662.795
Over-dominant	CT vs. TT + CC	1.062 (0.733–1.539)	0.749	666.897
Recessive	CC vs. CT + TT	22.328 (2.947–169.181)	0.003	645.789
Additive	C	1.792 (1.267–2.533)	<0.001	655.774

Abbreviations: OR—odds ratio, CI—confidence interval, AIC—Akaike information criterion, *p*-value—significance level.

**Table 5:** Binary logistic regression analysis of *TAS2R16* rs978739, rs1357949.

Model	Genotype/Allele	OR (95% CI)	*p*-Value	AIC
*TAS2R16* rs978739				
Codominant	CT vs. TT CC vs. TT	1.352 (0.928–1.968) 0.890 (0.443–1.789)	0.116 0.745	666.985
Dominant	CC + CT vs. TT	1.265 (0.884–1.812)	0.199	666.345
Over-dominant	CT vs. TT + CC	1.373 (0.953–1.976)	0.088	664.092
Recessive	CC vs. CT + TT	0.777 (0.395–1.530)	0.466	666.461
Additive	C	1.107 (0.834–1.467)	0.482	666.505
*TAS2R16* rs1357949				
Codominant	AG vs. AA GG vs. AA	0.914 (0.626–1.334) 0.909 (0.490–1.686)	0.641 0.762	668.753
Dominant	GG + AG vs. AA	0.913 (0.637–1.308)	0.620	666.753
Over-dominant	AG vs. AA + GG	0.930 (0.648–1.335)	0.694	666.845
Recessive	GG vs. AG + AA	0.950 (0.527–1.713)	0.865	666.970
Additive	G	0.939 (0.715–1.233)	0.650	666.794

Abbreviations: OR—odds ratio, CI—confidence interval, AIC—Akaike information criterion, *p*-value—significance level.

**Table 6:** Frequency of genotypes and alleles of the *TAS2R16* rs860170 in the females of the MS and control groups.

SNP	MS Group *n* (%) (*n* = 111)	Control Group *n* (%) (*n* = 169)	*p*-Value
*TAS2R16* rs860170			
Genotype			
TT	32 (28.8)	68 (40.2)	<0.001
CT	69 (62.2)	100 (59.2)	
CC	10 (9) ^1^	1 (0.6) ^1^	
Total	111 (100)	169 (100)	
Allele			
T	133 (59.9)	236 (69.8)	0.010
C	89 (40.1))	102 (30.2)	

^1^ *p* ≤ 0.001 (CC vs. CT + TT); Abbreviations: SNP—single nucleotide polymorphisms; *p*-value—significance level; The Chi-square (χ^2^) test was used.

**Table 7:** Frequency of *TAS2R16* rs978739 and rs1357949 genotypes and alleles females of the MS and control groups.

SNP	MS Group *n* (%) (*n* = 111)	Control Group *n* (%) (*n* = 169)	*p*-Value
*TAS2R16* rs978739			
Genotype			
TT	48 (43.2)	87 (51.5)	0.341
CT	53 (47.7)	66 (39.1)	
CC	10 (9)	16 (9.5)	
Total	111 (100)	169 (100)	
Allele			
T	149 (67.1)	240 (71)	0.328
C	73 (32.9)	98 (29)	
*TAS2R16* rs1357949			
Genotype			
AA	50 (45)	76 (45)	0.979
AG	50 (45)	75 (44.4)	
GG	11 (9.9)	18 (10.7)	
Total	111 (100)	169 (100)	
Allele			
A	150 (67.6)	227 (67.2)	0.920
G	72 (32.4)	111 (32.8)	

Abbreviations: SNP—single nucleotide polymorphisms; *p*-value—significance level; The Chi-square (χ^2^) test was used.

**Table 8:** Binary logistic regression analysis of *TAS2R16* rs860170 in females.

Model	Genotype/Allele	OR (95% CI)	*p*-Value	AIC
Codominant	CT vs. TT CC vs. TT	1.466 (0.872–2.467) 21.250 (2.607–173.207)	0.149 0.004	364.641
Dominant	CC + CT vs. TT	1.662 (0.995–2.776)	0.052	374.211
Over-dominant	CT vs. TT + CC	1.134 (0.694–1.852)	0.617	377.810
Recessive	CC vs. CT + TT	16.634 (2.098–131.870)	0.008	364.750
Additive	C	1.995 (1.259–3.160)	0.003	369.019

Abbreviations: OR—odds ratio, CI—confidence interval, AIC—Akaike information criterion, *p*-value—significance level.

**Table 9:** Binary logistic regression analysis of *TAS2R16* rs978739, rs1357949 in females.

Model	Genotype/Allele	OR (95% CI)	*p*-Value	AIC
*TAS2R16* rs978739				
Codominant	CT vs. TT CC vs. TT	1.455 (0.879–2.411) 1.133 (0.477–2.691)	0.145 0.778	377.913
Dominant	CC + CT vs. TT	1.393 (0.860–2.254)	0.178	376.237
Over-dominant	CT vs. TT + CC	1.426 (0.879–2.314)	0.151	375.993
Recessive	CC vs. CT + TT	0.947 (0.413–2.169)	0.897	378.044
Additive	C	1.201 (0.832–1.733)	0.329	377.107
*TAS2R16* rs1357949				
Codominant	AG vs. AA GG vs. AA	1.013 (0.611–1.680) 0.929 (0.405–2.132)	0.959 0.862	380.018
Dominant	GG + AG vs. AA	0.997 (0.616–1.613)	0.990	378.061
Over-dominant	AG vs. AA + GG	1.027 (0.635–1.663)	0.913	378.049
Recessive	GG vs. AG + AA	0.923 (0.418–2.036)	0.842	378.021
Additive	G	0.981 (0.682–1.412)	0.919	378.050

Abbreviations: OR—odds ratio, CI—confidence interval, AIC—Akaike information criterion, *p*-value—significance level.

**Table 10:** Frequency of genotypes and alleles of the rs860170 in the males of the MS and control groups.

SNP Genotypes/Alleles	MS Group *n* (%) (*n* = 107)	Control Group *n* (%) (*n* = 96)	*p*-Value
Genotype			
TT			
CT	31 (29)	32 (33.3)	0.036
CC	69 (64.5)	64 (66.7)	
Total	7 (6.5)	0 (0)	
Allele	107 (100)	96 (100)	
T			
C	131 (61.2)	128 (66.7)	0.254
	83 (38.8)	64 (33.3)	

Abbreviations: SNP—single nucleotide polymorphisms, *p*-value—significance level.

**Table 11:** Frequency of genotypes and alleles of the *TAS2R16* rs978739, rs1357949 in males.

SNP	MS Group *n* (%) (*n* = 107)	Control Group *n* (%) (*n* = 96)	*p*-Value
*TAS2R16* rs978739			
Genotype			
TT	56 (52.3)	55 (57.3)	0.461
CT	46 (43)	34 (35.4)	
CC	5 (4.7)	7 (7.3)	
Total	107 (100)	96 (100)	
Allele			
T	158 (73.8)	144 (75)	0.788
C	56 (26.2)	48 (25)	
*TAS2R16* rs1357949			
Genotype			
AA	52 (48.6)	42 (43.8)	0.772
AG	44 (41.1)	44 (45.8)	
GG	11 (10.3)	10 (10.4)	
Total	107 (100)	96 (100)	
Allele			
A	148 (69.2)	128 (66.7)	0.591
G	66 (30.8)	64 (33.3)	

Abbreviations: SNP—single nucleotide polymorphisms; *p*-value—significance level; The Chi-square (χ^2^) test was used.

**Table 12:** Binary logistic regression analysis of *TAS2R16* rs860170, rs978739, and rs1357949 in males.

Model	Genotype/Allele	OR (95% CI)	*p*-Value	AIC
*TAS2R16* rs860170				
Codominant	CT vs. TT CC vs. TT	1.113 (0.611–2.027) -	0.727 NA	275.510
Dominant	CC + CT vs. TT	1.226 (0.676–2.223)	0.503	282.372
Over-dominant	CT vs. TT + CC	0.908 (0.508–1.622)	0.744	282.715
Recessive	CC vs. CT + TT	NA	NA	273.632
Additive	C	1.505 (0.879–2.578)	0.136	280.575
*TAS2R16* rs978739				
Codominant	CT vs. TT CC vs. TT	1.329 (0.745–2.370) 0.720 (0.210–2.344)	0.336 0.565	283.267
Dominant	CC + CT vs. TT	1.222 (0.702–2.127)	0.479	282.320
Over-dominant	CT vs. TT + CC	1.375 (0.780–2.242)	0.271	281.603
Recessive	CC vs. CT + TT	0.623 (0.191–2.033)	0.433	282.197
Additive	C	1.066 (0.676–1.679)	0.784	282.746
*TAS2R16* rs1357949				
Codominant	AG vs. AA GG vs. AA	0.808 (0.451–1.447) 0.888 (0.344–2.293)	0.473 0.807	284.304
Dominant	GG + AG vs. AA	0.823 (0.473–1.431)	0.489	282.343
Over-dominant	AG vs. AA + GG	0.825 (0.473–1.440)	0.499	282.364
Recessive	GG vs. AG + AA	0.985 (0.399–2.435)	0.975	282.820
Additive	G	0.892 (0.588–1.354)	0.592	282.534

Abbreviations: OR—odds ratio, CI—confidence interval, AIC—Akaike information criterion, *p*-value—significance level.

**Table 13:** Haplotype association of *TAS2R16* rs860170, rs978739, rs1357949 with the predisposition to MS occurrence.

Haplotype	*TAS2R16* rs860170	*TAS2R16* rs978739	*TAS2R16* rs1357949	Frequency (%)		OR (95% CI)	*p*-Value
				Control	MS		
1	C	T	A	30.78	27.2	1.00	–
2	T	T	G	31.28	19.15	0.76 (0.49–1.20)	0.240
3	T	C	A	25.45	23.34	1.04 (0.67–1.63)	0.860
4	T	T	A	10.39	14.84	1.52 (0.84–2.73)	0.170
5	T	C	G	1.55	3.22	1.33 (0.39–4.58)	0.650
6	C	C	A	0.36	2.97	12.51 (1.59–104.12)	0.020
Rare	*	*	*	NA	NA	6.85 (0.47–98.99)	0.160

Abbreviations: MS—multiple sclerosis; OR—odds ratio; CI—confidence interval; *p*-value—significance level. *—All haplotypes with less than 1% frequencies were grouped together and labeled as “rare” haplotypes.

**Table 14:** Haplotype association of *TAS2R16* rs860170, rs978739, rs1357949 with the predisposition to exudative AMD occurrence in females.

Haplotype	*TAS2R16* rs860170	*TAS2R16* rs978739	*TAS2R16* rs1357949	Frequency (%)		OR (95% CI)	*p*-Value
				Control	MS		
1	C	T	A	29.83	24.93	1.00	–
2	T	T	G	31.26	16.33	0.73 (0.39–1.38)	0.330
3	T	C	A	27.41	22.87	1.03 (0.56–1.88)	0.930
4	T	T	A	9.92	15.23	1.87 (0.80–4.34)	0.150
5	T	C	G	1.24	5.48	3.94 (0.85–18.28)	0.081
rare	*	*	*	-	NA	7.76 (0.64–94.25)	0.110

Abbreviations: MS—multiple sclerosis; OR—odds ratio; CI—confidence interval; *p*-value—significance level. *—All haplotypes with less than 1% frequencies were grouped together and labeled as “rare” haplotypes.

**Table 15:** Haplotype association of *TAS2R16* rs860170, rs978739, rs1357949 with the predisposition MS occurrence in males.

Haplotype	*TAS2R16* rs860170	*TAS2R16* rs978739	*TAS2R16* rs1357949	Frequency (%)		OR (95% CI)	*p*-Value
				Control	MS		
1	C	T	A	32.57	29.33	1.00	–
2	T	T	G	31.15	22.41	0.87 (0.44–1.72)	0.690
3	T	C	A	22.05	24.55	1.27 (0.62–2.58)	0.520
4	T	T	A	11.28	14.25	1.43 (0.60–3.37)	0.420
5	T	C	G	2.19	0	0.20 (0.00–91.98)	0.610
Rare	*	*	*	-	-	1.79 (0.10–32.48)	0.690

Abbreviations: MS—multiple sclerosis; OR—odds ratio; CI—confidence interval; *p*-value—significance level. *—All haplotypes with less than 1% frequencies were grouped together and labeled as “rare” haplotypes.

## Data Availability

The data will be sent upon request.
